# Functional Characterization of the Nep1-Like Protein Effectors of the Necrotrophic Pathogen – *Alternaria brassicae*

**DOI:** 10.3389/fmicb.2021.738617

**Published:** 2021-10-26

**Authors:** Deepak Duhan, Shivani Gajbhiye, Rajdeep Jaswal, Ravindra Pal Singh, Tilak Raj Sharma, Sivasubramanian Rajarammohan

**Affiliations:** ^1^Agricultural Biotechnology Division, National Agri-Food Biotechnology Institute, Mohali, India; ^2^Nutritional Biotechnology Division, National Agri-Food Biotechnology Institute, Mohali, India; ^3^Indian Council of Agricultural Research, Division of Crop Science, Krishi Bhavan, New Delhi, India

**Keywords:** *Alternaria brassicae*, necrotroph, NLP, necrosis, effectors

## Abstract

*Alternaria brassicae* is an important necrotrophic pathogen that infects the *Brassicaceae* family. *A. brassicae*, like other necrotrophs, also secretes various proteinaceous effectors and metabolites that cause cell death to establish itself in the host. However, there has been no systematic study of *A. brassicae* effectors and their roles in pathogenesis. The availability of the genome sequence of *A. brassicae* in public domain has enabled the search for effectors and their functional characterization. Nep1-like proteins (NLPs) are a superfamily of proteins that induce necrosis and ethylene biosynthesis. They have been reported from a variety of microbes including bacteria, fungi, and oomycetes. In this study, we identified two NLPs from *A. brassicae* viz. AbrNLP1 and AbrNLP2 and functionally characterized them. Although both AbrNLPs were found to be secretory in nature, they localized differentially inside the plant. AbrNLP2 was found to induce necrosis in both host and non-host species, while AbrNLP1 could not induce necrosis in both species. Additionally, AbrNLP2 was shown to induce pathogen-associated molecular pattern (PAMP)-triggered immunity in both host and non-host species. Overall, our study indicates that AbrNLPs are functionally and spatially (subcellular location) distinct and may play different but important roles during the pathogenesis of *A. brassicae*.

## Introduction

Plant pathogens secrete various proteins, secondary metabolites, and other small molecules to colonize the host by suppressing host defenses (Kamoun, 2006; Lindeberg et al., 2008). In contrast, many pathogens also produce proteins that trigger cell death and associated responses in the host. Various filamentous fungal and oomycetes pathogens can induce cell death in the plants through the secretion of cell death inducing proteins/effectors (Torto et al., 2003; Gijzen and Nurnberger, 2006; Amaro et al., 2017). Cell death plays a central role in plant-pathogen interactions either by restricting pathogen growth (apoptotic-like cell death in case of biotrophs) or by providing a nutrient source for the proliferation of the pathogen (necrotic cell death in case of necrotrophs). Many well-known necrotrophic pathogens such as *Botrytis cinerea*, *Sclerotinia sclerotiorum*, *Alternaria brassicae* have been shown to induce necrotic cell death in the host and effective induction of necrotic cell death leads to susceptibility (Lyu et al., 2016; Zhu et al., 2017; Mandal et al., 2018).

The genus *Alternaria* contains many important plant pathogens causing diseases in agronomically important cereal, vegetable, oilseed, and fruit crops. These species are known to be one of the major producers of host-specific toxins and metabolites that cause necrotic cell death and help in disease progression. *Alternaria* spp. produce chemically diverse secondary metabolites that range from low molecular weight secondary metabolites to peptides. These toxins can be host-specific as well as non-specific. Tenuazonic acid, tentoxin, and brefeldin A are some examples of non-specific toxins secreted by *Alternaria* spp., which are phytotoxic in nature (Meronuck et al., 1972; Fujiwara et al., 1988). Host-specific toxins (HSTs) such as AK-toxin, AF-toxin, ACT-toxin, AM-toxin, AAL-toxin are secreted by various pathotypes of *A. alternata* (Tanaka and Tsuge, 2000; Hatta et al., 2002; Thomma, 2003). HSTs have been shown to be the major pathogenicity factors in *A. alternata* and disruption of the genes coding for these HSTs leads to loss of pathogenicity. However, very little is known about the molecular mechanisms underlying pathogenesis of other *Alternaria* species such as *Alternaria brassicae* and *Alternaria brassicicola*. Destruxin B was considered to be an HST of *A. brassicae*, but later studies proved that Destruxin B only aids in the infection process of *A. brassicae* (Parada et al., 2008). Recent studies have revealed that necrotrophic pathogens also secrete small proteinaceous effectors that can function as pathogenicity factors. Some prominent examples of proteinaceous necrotrophic effectors that induce necrotic cell death include PtrToxA, SnToxA, BcCFEM1, BcXYG1, and the NEP-like proteins in various necrotrophs (Ciuffetti et al., 2010; Dallal Bashi et al., 2010; Arya et al., 2020).

Nep1-like proteins (NLPs) are a group of proteins identifiable by the presence of a common necrosis-inducing NPP1 domain (Fellbrich et al., 2000, 2002). NLPs constitute a superfamily of proteins, which are present in both prokaryotes and eukaryotes (Pemberton and Salmond, 2004; Bae et al., 2006; Gijzen and Nurnberger, 2006). The founding member of this family is a 24-kDa protein (Nep1) that was identified in *Fusarium oxysporum* that could cause necrosis and induce ethylene biosynthesis in dicots (Bailey, 1995). The NPP1 domain present in all NLPs is characterized by a conserved heptapeptide motif—GHRHDWE, and N-terminal conserved cysteine residues. Based on the number of cysteine residues, NLPs are classified as type I (two conserved cysteine residues) or type II (four conserved cysteine residues) (Gijzen and Nurnberger, 2006). Most NLPs identified in oomycetes and fungi trigger cell death and also act as pathogen-associated molecular patterns (PAMPs), thereby activating PAMP-triggered immunity (PTI) (Veit et al., 2001; Qutob et al., 2002, 2006). However, the role of NLPs in virulence is conflicting with NLPs being dispensable for virulence in *Zymoseptoria tritici* and *B. cinerea* and known to accelerate disease and pathogen growth in *Colletotrichum coccodes*, *Phytophthora capsici* and *Pythium* species (Motteram et al., 2009; Cuesta Arenas et al., 2010; Feng et al., 2014). However, there have been no studies on the identification or functional characterization of NLPs from the genus of *Alternaria*.

The current study was undertaken to identify and functionally characterize the NLPs in *A. brassicae*, a notorious necrotrophic pathogen that mainly infects the *Brassicaceae* family and also some plant species from other families (Ansari et al., 1990). Specifically, we aimed to: (1) identify the NLPs in *A. brassicae* and their phylogenetic relationship with other known NLPs, (2) study the temporal expression pattern in the natural host—*Brassica juncea*, (3) determine their ability to cause necrosis in host as well as non-host species, (4) identify their subcellular localization, and (5) analyze if the NLPs in *A. brassicae* also acted as PAMPs and induced PTI in the host.

## Materials and Methods

### Fungal Strains, Plants, and Culture Conditions

The fungal strain, *A. brassicae* J3 (Rajarammohan et al., 2017) was used for the infection assays in the expression analysis. The strain was grown on potato dextrose agar (PDA, pH adjusted to 7.0 using 1 N NaOH) plates (90 mm) at 22°C for 15 days under 12-h/12-h light/dark cycle. For the transient expression assays, *Nicotiana benthamiana* wild type plants grown at 25°C with photoperiod of 10 h/14 h light/dark were used. Similarly, *B. juncea* var. Varuna was grown at 25°C with photoperiod of 10 h Light/14 h dark and used for the infection and infiltration assays. A 5–6 week old plants of *N. benthamiana* and *B. juncea* were used for all the experiments.

### Identification and *in silico* Analysis of *Alternaria brassicae* Nep1-Like Proteins

NLP genes in *A. brassicae* were identified by a BLAST search of the *A. brassicae* proteome using the NPP1 domain (Pfam: PF05630) as a query. Signal peptides were predicted using SignalP 5 (Almagro Armenteros et al., 2019). Phylogenetic analysis was performed using MEGA X (Kumar et al., 2018). Since *A. brassicae* belongs to the class of *Dothideomycetes* and the division of *Ascomycota*, protein sequences of other *Ascomycetes* members were retrieved using a similar BLAST search as described above against the nr database (Supplementary Table 1). Protein sequences were aligned using MUSCLE and outliers with too many gaps were removed from the analysis. The WAG + G substitution model was selected since it had the least Bayesian Information Criterion (BIC) value among the 56 amino acid substitution models tested. A phylogenetic tree was constructed using the WAG + G model in MEGA X and the final tree was visualized in iTOL (Letunic and Bork, 2021).

### Gene Expression Analysis

Fungal cultures were established and plant infection assays were carried out as described earlier (Mandal et al., 2018). Briefly, 15-day old PDA plates were used to prepare spore suspension for drop inoculations. A 15 μl droplets of the spore suspension (concentration of 3 × 10^3^ conidia/ml) was placed on the leaves of 5–6-week old *B. juncea* plants. The plants were then maintained at > 90% relative humidity to enable infection. Leaves were collected at two and 4 days post inoculation (2 and 4 dpi). Fifteen-day old *A. brassicae* mycelia growing on PDA was also harvested (*in vitro* sample). Total RNA was extracted from 2–3 leaves collected from three individual plants in each experiment using the RNeasy plant mini kit according to the manufacturer’s recommendation (Qiagen, Gaithersburg, MD, United States). First-strand cDNA was synthesized from 1 μg of total RNA using PrimeScript 1st-Strand cDNA Synthesis Kit (Takara Bio, Japan) as per manufacturer’s protocol. PCRs were carried out using the standard setting in a CFX Connect Real-Time PCR System (BIO-RAD, CA, United States). The housekeeping gene AbrActin was used as the endogenous control. Further, to calculate the relative expression levels, the dCt values of the infected samples were normalized to the *in vitro* sample (*A. brassicae* mycelia on PDA). Relative expression values were calculated using the delta delta Ct method (Pfaffl, 2001). All expression experiments were carried out in three biological replicates. Every biological replicate also consisted of three technical replicates. Sequences of primers used in qPCR have been listed in Supplementary Table 2.

### Yeast Secretion Trap Assay

Experimental validation of the secretory nature of the AbrNLPs was done using yeast secretion trap (YST) assay (Plett et al., 2011). The pSUC2-GW vector carries a truncated invertase (SUC2) lacking its signal peptide. The *AbrNLP* and *AvrPiz-t* cDNAs were cloned upstream (in-frame) of the truncated SUC2 gene. Yeast strain YTK12, which is SUC2 negative, was transformed using the Fast Yeast Transformation kit (G-Biosciences, MO, United States). All transformants were selected on a yeast minimal medium without tryptophan (6.7 g/L Yeast Nitrogen Base without amino acids, 0.7 g/L tryptophan dropout supplement, 20 g/L glucose, 15 g/L agar, pH 5.6). To check for secretion, a 10-fold serial dilution of overnight yeast cultures were made. Three dilutions (10^0^, 10^–1^, and 10^–2^) of each construct and SUC2^–^ cells were plated onto YPSA medium (1% yeast extract, 2% peptone, 2% sucrose and 1 μg/ml antimycin A after autoclaving, pH 6.5). The untransformed YTK12 strain, which is unable to grow on a sucrose medium, was used as a negative control.

### Transient Expression of AbrNLP1 and AbrNLP2

#### Molecular Cloning and Sequencing

The *AbrNLP* genes (with signal peptide and without stop codon) were amplified from cDNA of *A. brassicae* infected leaves of *B. juncea* and cloned in the pENTR entry vector. The entry clone was then Sanger sequenced to confirm the cDNA sequences of *AbrNLPs*. The entry clones were then transferred to different destination vectors using LR clonase II (Gateway cloning technology) for functional assays. Three destination vectors were used viz. pGWB408, pGWB441, and pDEST17. Positive clones of the destination vectors were confirmed using colony PCR and restriction digestion. The confirmed vectors were then mobilized into the *Agrobacterium tumefaciens* GV3101 strain.

#### Cell Death Assays

Agroinfiltration assays were carried out as described earlier (Kanneganti et al., 2007). Primary cultures of pGWB408-AbrNLP1 and pGWB408-AbrNLP2 grown overnight were used for inoculating secondary cultures, which were grown till they reached an OD_600_ of 0.8–1. The pellets from the secondary cultures were resuspended in the resuspension solution (MES—10 mM, MgCl_2_—10 mM, and Acetosyringone—200 μM). The resuspended cultures were incubated at room temperature (25°C) for 3–4 h prior to infiltration in 5–6 weeks old *Nicotiana benthamiana* and *B. juncea* leaves. The experiments were repeated at least three times with 4–6 leaves infiltrated in each experiment for each construct. Cell death was observed visually 3–4 days post infiltration.

#### Subcellular Localization

The AbrNLPs were cloned into the pGWB441 vector to have a C-terminal yellow fluorescent protein (YFP) fusion using Gateway Cloning. Primary cultures of pGWB441-AbrNLP1 and pGWB441-AbrNLP2 grown overnight were used for inoculating secondary cultures, which were grown till they reached an OD_600_ of 0.8–1. The pellets from the secondary cultures were resuspended in the resuspension solution (MES—10 mM, MgCl2—10 mM, and Acetosyringone—200 μM). The resuspended cultures were incubated at room temperature (25°C) for 3–4 h prior to infiltration in 5–6 weeks old *N. benthamiana* leaves. The subcellular localization of YFP-tagged proteins was examined 2–3 days post infiltration using a Carl Zeiss confocal microscope (LSM880). For visualization of the nucleus and cell wall, the leaves were immersed in 4′,6-diamidino-2-phenylindole (DAPI) solution (10 μg/ml) and Propidium Iodide solution (10 μg/ml) sequentially for 15 min in each solution before imaging. Excitation of the corresponding leaf areas took place at 514 nm for YFP (Filter Set 38 HE, Carl Zeiss AG), at 405 nm for DAPI (Filter Set 49 HE, Carl Zeiss AG), and at 561 nm for PI (Filter Set 43 HE, Carl Zeiss AG).

#### Heterologous Expression and Purification

For protein expression, the entry clone was mobilized into the pDEST17 destination vector. The pDEST17 vector is an expression vector with a N-terminal 6xHis tag and a T7 terminator region flanking the attR1 and attR2 Gateway recombination sites. Positive clones were transformed into BL21(DE3)pLysS competent cells. The cultures were induced with 0.4 mM IPTG at 22°C. Following expression, the cells were harvested and cell lysis was carried out by chemical, enzymatic and mechanical treatment. For chemical lysis, cells were suspended in a lysis buffer (containing 100 mM NaCl, 50 mM Tris buffer of pH8.0, 200 μl Barry’s buffer and 2 mM PMSF). The cell suspensions were then subjected to lysis with 200 μg/g lysozyme for 30 min followed by treatment with DNase I for 1 h at room temperature. The lysates were then sonicated with 40% amplitude, 10 s ON/30 s OFF cycle for 3 min on ice using a Sonics Vibra-cell (VC505) sonicator, followed by centrifugation at 10,000 *g* for 40 min at 4°C. The supernatant was then subject to Ni-NTA purification using Ni Sepharose High-Performance resin (Cytiva Lifesciences, Marlborough, MA, United States). The eluted purified protein was desalted to remove excess imidazole using centrifugal filter columns with a 3 kDa cutoff (Amicon Ultra-0.5 ml Centrifugal filters, Merck). The purified protein was further used for cytotoxicity and secreted peroxidase (sPOX) assays.

#### Cytotoxicity of AbrNLP2 and Secreted Peroxidase Assay

Purified AbrNLP2 was directly infiltrated into the leaves of 5–6-week old *N. benthamiana* and *B. juncea* plants to check for cytotoxicity. The sPOX assay was carried out as described earlier with minor modifications (Mott et al., 2018). Briefly, 5 mm leaf disks were excised from *N. benthamiana* and *B. juncea* plants and washed with distilled water for 2 h with agitation in a 96 well plate. After washing, water was carefully removed out while minimizing the damage to the leaf disks. 100 μl of purified AbrNLP2 protein was added in the designated wells while remaining wells filled with buffer and incubated for 20 h with agitation. After incubation, the leaf disks were carefully removed from the wells and 50 μl of 5-aminosalicylic acid (1 mg/ml) supplemented with 0.01% H_2_O_2_ was added in each well. After 3 min, 20 μl of 2N NaOH was added to each well to stop the reaction and absorbance was measured at OD_600_ in a SpectraMax i3x spectrophotometer (Molecular Devices). A heteroscedastic (between two samples of unequal variances) single tailed *t*-test was conducted to determine if there were significant differences in the sPOX activities of the buffer-treated samples and AbrNLP2-treated samples.

## Results

### The Genome of *Alternaria brassicae* Encodes Two Nep1-Like Proteins

**FIGURE 1:**
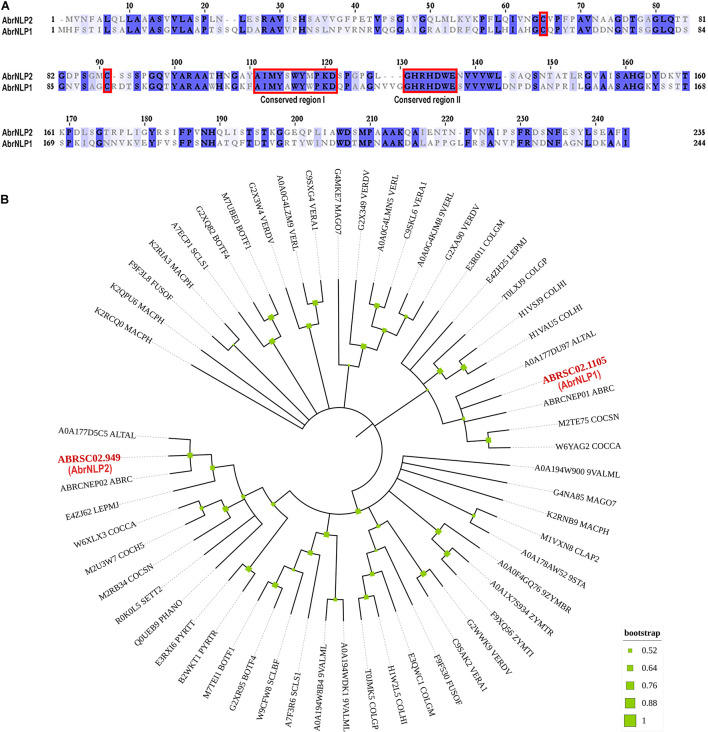
Sequence characteristics of AbrNLPs and their phylogenetic relationship. **(A)** Multiple sequence alignment of NLPs from *A. brassicae*, the two conserved cysteines and conserved regions I and II that classify AbrNLPs as type I NLPs, are highlighted in red. **(B)** Phylogenetic relationship of AbrNLP1 and AbrNLP2 compared to the NLPs of other pathogens was inferred by using the Maximum Likelihood method. The bootstrap consensus tree was inferred from 1,000 replicates. Branches corresponding to partitions reproduced in less than 50% bootstrap replicates were collapsed. A discrete Gamma distribution was used to model evolutionary rate differences among sites [5 categories (+ G, parameter = 1.4565)]. All positions with less than 95% site coverage were eliminated, i.e., fewer than 5% alignment gaps, missing data, and ambiguous bases were allowed at any position.

To identify NLPs in the genome of *A. brassicae*, we searched the whole protein dataset using the NPP1 domain as a query. We found two genes that contained the NPP1 domain viz. ABRSC02.1105 and ABRSC02.949 (hereafter referred to as AbrNLP1 and AbrNLP2 respectively) (GenBank accession numbers: MZ783062 and MZ783063). The two AbrNLPs identified were highly diverged sharing only ∼40% similarity (Figure 1A). Additionally, both the AbrNLPs contained the two conserved cysteine residues and the heptapeptide motif (GHRHDWE), which classified them as a type I NLP (Gijzen and Nurnberger, 2006). In order to reconstruct the relationship between AbrNLPs and the NLPs identified in other *Ascomycetes*, a phylogenetic analysis was carried out. As expected the AbrNLP1 clustered along with NLP1 homologs from other *Alternaria* species, while AbrNLP2 clustered together with the NLP2 homologs (Figure 1B).

### AbrNLP1 and AbrNLP2 Both Are Induced Upon Infection in *Brassica juncea*

**FIGURE 2:**
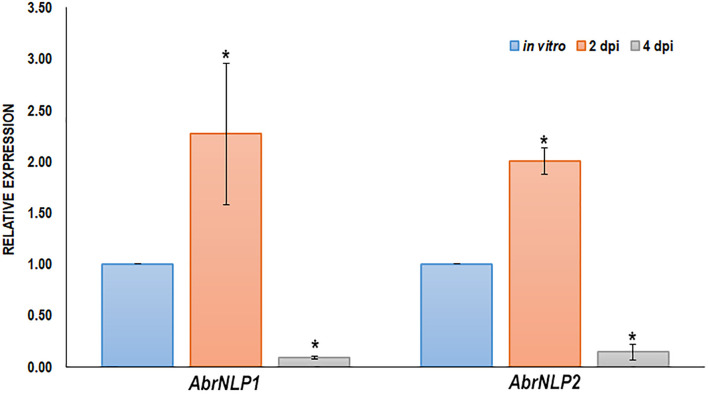
Expression of *AbrNLPs* during infection of *B. juncea*. Relative expression analysis of AbrNLP1 and AbrNLP2 in infected *B. juncea* (natural host) leaves 2 and 4 days post inoculation with respect to plate-grown fungi (*in vitro*). AbrACTIN was used as an endogenous control. The mean values (± standard deviation) of three biological replicates are shown. *P < 0.05 by Mann–Whitney *U*-test.

The expression of AbrNLP1 and AbrNLP2 was studied *in vitro* (on PDA plates) and over the course of inoculation in the natural host of *A. brassicae* i.e., *B. juncea*. Previous studies on the infection of *A. brassicae* have identified two critical time points viz. 2 and 4 days post inoculation wherein it represented the time points before and after the mycelia penetrates the host tissue (Mandal et al., 2019). Therefore, we studied the expression of AbrNLP1 and AbrNLP2 at these time points. We found that both AbrNLP1 and AbrNLP2 are expressed *in vitro* i.e., in growing fungal mycelia on solid media. However, they are both upregulated during the infection process at 2 dpi and thereafter are downregulated at 4 dpi when infection is established (Figure 2).

### AbrNLP1 and AbrNLP2 Contain Bonafide Signal Peptides and Are Secreted Outside the Cell

**FIGURE 3:**
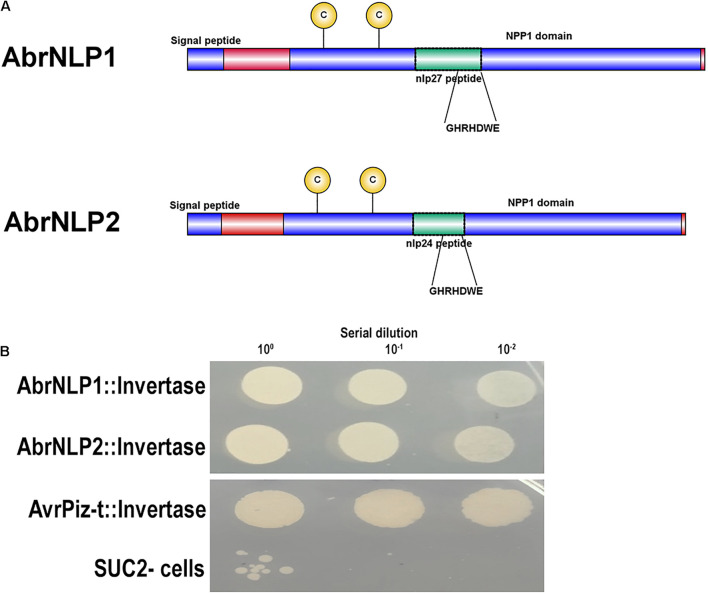
AbrNLP1 and AbrNLP2 are secreted proteins as determined by the YST assay. **(A)** Schematic diagram of AbrNLP1 and AbrNLP2 indicating the signal peptides, NPP1 domains, and nlp24 and nlp27 conserved regions containing the heptapeptide motif GHRHDWE. **(B)** Yeast strain SUC2^–^ was transformed with AbrNLP1:Invertase, AbrNLP2:Invertase, and AvrPiz-t:Invertase, and a serial dilution of overnight cultures of the transformed strains were plated on medium containing sucrose with antimycin (1 μg/ml). Untransformed SUC2^–^ was used as a negative control. AvrPiz-t, a known secretory protein from *M. oryzae*, was used as a positive control for the assay.

Both the proteins, AbrNLP1 and AbrNLP2 were predicted to have an N-terminal signal peptide and most likely to be secreted (Figure 3A). Further, AbrNLP1 and AbrNLP2 were predicted to be effectors by the EffectorP 2.0 tool (Sperschneider et al., 2018a). In order to confirm the secretion of AbrNLP1 and AbrNLP2, a yeast invertase secretion assay was carried out. Colonies that contain the invertase fusion proteins with a functional signal peptide will only be able to grow on the selection media. A serial dilution of the liquid cultures was plated on the sucrose selection medium wherein constructs expressing both AbrNLP1 and AbrNLP2 fusion proteins grew, indicating that the N-terminal signal peptide resulted in the secretion of these proteins outside the cell (Figure 3B). Additionally, we also plated the SUC2^–^ cells as a negative control (that did not grow or showed only minimal growth at highest dilution) and AvrPiz-t, a known secretory protein from *Magnaporthe oryzae* (Park et al., 2012) as a positive control, which grew on the selection medium (Figure 3B).

### AbrNLP2 Induces Necrotic Cell Death While AbrNLP1 Induces Only Chlorosis in *Nicotiana benthamiana* and *Brassica juncea*

**FIGURE 4:**
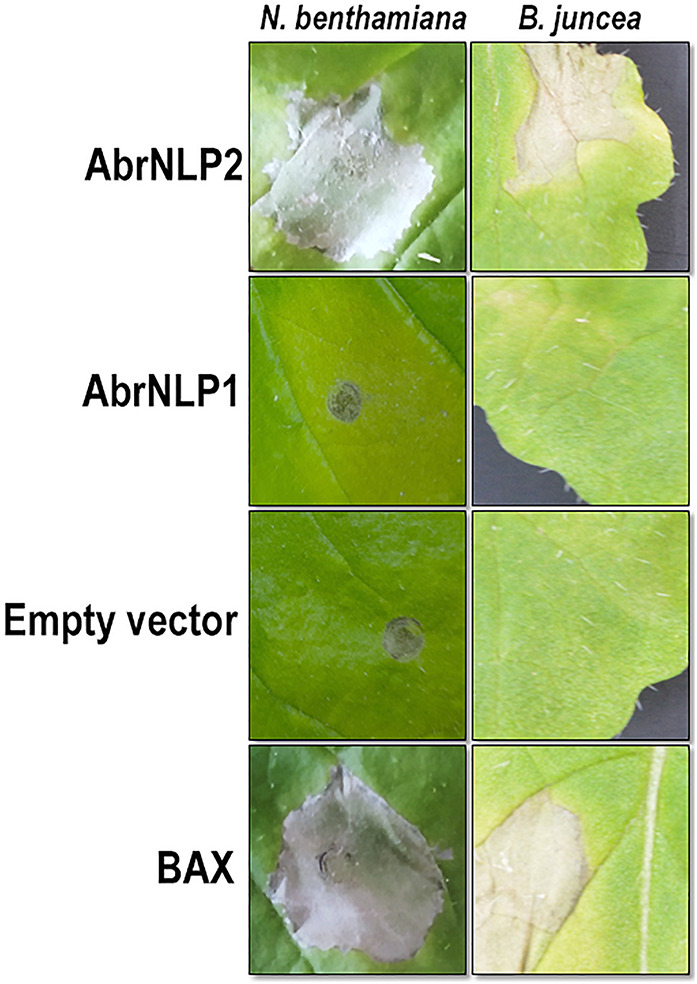
Agroinfiltration of AbrNLP1 and AbrNLP2 in *N. benthamiana* and *B. juncea*. Transient expression of AbrNLP1 and AbrNLP2 in both host and non-host species. BAX was used as a positive control for induction of cell death and empty vector (pGWB408) was used as a negative control.

To check AbrNLPs for their ability to induce necrosis, the full-length genes (along with their signal peptide) were transiently expressed in *N. benthamiana* and *B. juncea* (natural host). BAX (Bcl2-associated X), known to induce cell death in plants, was used as a positive control. AbrNLP1 could not induce cell death in *N. benthamiana* or *B. juncea* even after 5–7 days of agroinfiltration. However, *N. benthamiana* leaves infiltrated with AbrNLP1 developed chlorosis after 3 days of infiltration, which was absent in the regions infiltrated with empty vector controls (Figure 4). AbrNLP2 induced necrotic cell death 2 days after agroinfiltration in *N. benthamiana* and *B. juncea*.

### Differential Localization of AbrNLP1 and AbrNLP2 in *Nicotiana benthamiana*

**FIGURE 5:**
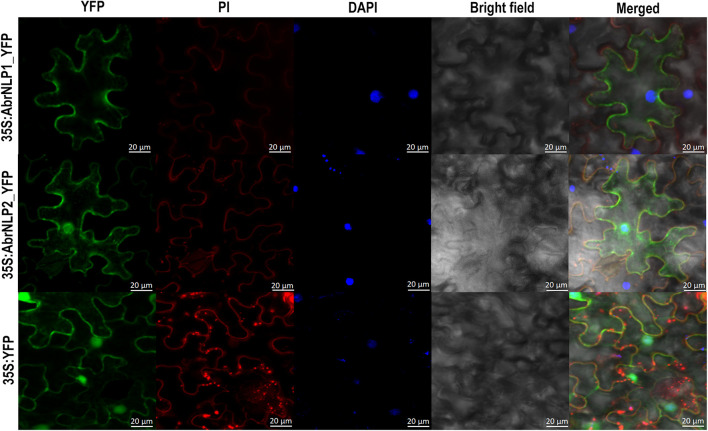
Subcellular localization of YFP-tagged AbrNLP1 and AbrNLP2 in *N. benthamiana*. Confocal microscopy images of transiently expressed C-terminal YFP-tagged AbrNLPs in *N. benthamiana* 72 hpi. Full-length YFP was used as a positive control. The leaf sections were stained with DAPI and PI to visualize the nucleus and plant cell wall respectively. Scale bar = 20 μm.

AbrNLP1 was predicted to be localized in the apoplast, while AbrNLP2 was predicted to be non-apoplastic by the ApoplastP tool (Sperschneider et al., 2018b). In order to experimentally determine the subcellular localization of AbrNLP1 and AbrNLP2, these proteins were transiently expressed in *N. benthamiana* with a C-terminal YFP tag. Full-length YFP under the 35S promoter was used as a positive control. Additionally, the leaves were stained using DAPI and PI to visualize the nucleus and plant cell wall. The AbrNLP1-YFP fusion proteins accumulated at the membrane or boundaries of the cell, and co-localized with PI, which stains the cell wall (Figure 5). Therefore, we can conclude that AbrNLP1 is localized at the plasma membrane/apoplastic space between the cell junctions. However, the AbrNLP2-YFP fusion proteins accumulated both within the cell and also at the plasma membrane. The YFP signals co-localized with both DAPI (staining the nucleus) and PI (staining the cell wall) indicating that AbrNLP2 is localized both within the cell and also in the membrane/apoplastic space (Figure 5).

### AbrNLP2 Induces PAMP-Triggered Immunity Responses in *Nicotiana benthamiana* and *Brassica juncea*

**FIGURE 6:**
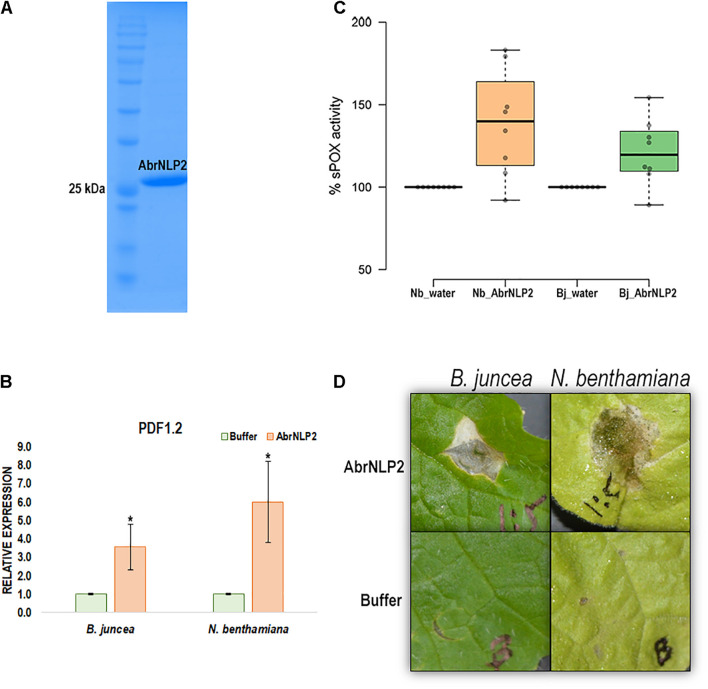
AbrNLP2 induces necrotic cell death and PTI responses. **(A)** Ni-NTA based purification of AbrNLP2. **(B)** Relative expression of PDF1.2 in AbrNLP2 and buffer treated leaf disks of *N. benthamiana* and *B. juncea*. TIPS41 was used as an endogenous control. *P < 0.05 by Mann–Whitney *U*-test. **(C)** %sPOX activity of *N. benthamiana* and *B. juncea* in response to purified AbrNLP2 as compared to water (control). **(D)** Cell death induced by purified AbrNLP2 in *N. benthamiana* and *B. juncea*.

Nep1-like proteins from various pathogens have been shown to be both cytotoxic as well as an inducer of PTI. Therefore, to check if AbrNLPs could also induce PTI responses in the host, we heterologously expressed AbrNLPs in *E. coli* and purified them (Figure 6A). However, AbrNLP1 was consistently found in the inclusion bodies in all the conditions and hence could not be purified. AbrNLP2 was purified and was used for both cytotoxic assays and a secreted peroxidase assay, which is an indicator of PTI response (Mott et al., 2018). The purified AbrNLP2 was used for the sPOX assay, wherein AbrNLP2 treated leaf disks of *N. benthamiana* and *B. juncea* had significantly higher sPOX activity than the buffer treated leaf disks (Nb: *p*-value = 0.00587, *t*-stat = −3.38; Bj: *p*-value = 0.0103, *t*-stat = −2.97) indicating that AbrNLP2 is also capable of inducing PTI (Figure 6C). Additionally, we also checked for the induction of the defense response gene PDF1.2 in leaf disks treated with AbrNLP2. We found that AbrNLP2 could significantly induce PDF1.2 expression in both *B. juncea* and *N. benthamiana* in comparison to the buffer treated leaf disks (Figure 6B). Purified AbrNLP2 protein could also induce necrotic cell death in both *N. benthamiana* and *B. juncea* within 2 days of infiltration (Figure 6D).

## Discussion

An emerging number of studies have shown that cell death-inducing proteins/effectors are required for pathogenicity, or contribute to the virulence of necrotrophic pathogens (Gijzen and Nurnberger, 2006; Ciuffetti et al., 2010; Lyu et al., 2016; Amaro et al., 2017; Zhu et al., 2017). Proteinaceous necrotrophic effectors have received less attention due to the gamut of earlier studies focusing on secondary metabolite toxins as the major pathogenicity factors in many of the necrotrophic pathogens (Cessna et al., 2000; Tanaka and Tsuge, 2000; Thomma, 2003). Recent genome sequencing efforts and functional studies have shown that proteinaceous effectors of necrotrophic pathogens have a larger role to play in the pathogenicity than previously surmised (Lyu et al., 2016; Derbyshire et al., 2017; Rajarammohan et al., 2019).

NLPs are a superfamily of proteins found in pathogenic bacteria, fungi, and oomycetes and have been implicated in their virulence and disease pathogenesis (Gijzen and Nurnberger, 2006). In the current work, we identified and characterized the NLPs in *A. brassicae*, an important pathogen belonging to the genus of *Alternaria*. We found that most species contained only two copies of the NLP genes within the genus of *Alternaria*, while some endophytes have only one (Supplementary Table 3). This is in contrast with oomycetes pathogens, which have an expanded repertoire of NLPs reaching up to 33 copies in *P. sojae* (Dong et al., 2012). Additionally, we found no correlation between the presence of the NLP proteins and the lifestyle of the fungal pathogens. However, most broad range necrotrophs such as *B. cinerea*, *S. sclerotiorum*, and *A. brassicae* possess only two copies of NLPs (Schouten et al., 2008; Oome et al., 2014).

Both the NLPs of *A. brassicae* were upregulated in the initial stages of infection, suggesting that AbrNLP1 and AbrNLP2 may have a role in pathogenesis. However, AbrNLP1 did not induce cell death *in N. benthamiana* or *B. juncea*. Moreover, AbrNLP1 did induce clear chlorosis in *N. benthamiana*, which may be due to weak induction of PTI in response to AbrNLP1. Most NLPs that have been identified in necrotrophs have a strong ability to induce necrosis. However, many non-cytotoxic NLPs have been found in hemibiotrophic and biotrophic pathogens (Oome et al., 2014). Therefore, it is possible that certain NLPs have a role that is independent of cytotoxicity. One such plausible role can be attributed to NLPs by its structural similarity to lectins (Ottmann et al., 2009). Lectins are defined by their ability to bind various carbohydrates such as β-1-3-glucans that are present in the cell wall of filamentous pathogens. Therefore, NLPs being structurally similar to lectins, may bind to cell wall glucans and thereby suppress the recognition of the pathogen by the host surveillance systems. However, this possibility needs further investigation.

Both the AbrNLPs contained bonafide signal peptides and were demonstrated to be secretory in nature using a yeast secretion trap assay. However, their subcellular localization in *N. benthamiana* was contrasting. While AbrNLP1 was associated with the plasma membrane and the apoplastic space, AbrNLP2 was found not only in the plasma membrane but also in the cytoplasm and nucleus. It is interesting to note that variation in the localization of NLPs within the same species has not been reported to date. However, different NLPs from different pathogens have been shown to have different subcellular locations. For example, NLPs from *B. cinerea* were associated with the nucleus and nuclear membrane (Schouten et al., 2008). A type II NLP from *F. oxysporum* was also localized to the cytoplasm using an immunogold-labeling technique (Bae et al., 2006). NLPs from the obligate biotroph *Plasmopara viticola* were also found to be localized within the cytoplasm and nucleus as is the case of AbrNLP2 (Schumacher et al., 2020). However, we could not distinguish whether AbrNLP2 is transported into the nucleus or it accumulates at the nuclear membrane (due to its membrane binding affinity). Moreover, the subcellular localization of the NLPs could not be correlated to the cell death inducing ability, since NLPs from *B. cinerea* and *A. brassicae* have been shown to cause cell death in the host even though they localize to both the membrane/apoplastic space and in the nucleus and cytoplasm. In contrast, NLPs from *P. viticola* localize intracellularly but do not cause any cell death.

AbrNLP2 was also shown to induce PTI in both non-host (*N. benthamiana*) and host (*B. juncea*) using the secreted peroxidase assay. The quantum of PTI was higher in the non-host, *N. benthamiana*, which is concordant with the literature that PTI responses against a pathogen are stronger in a non-host vs. a natural host (Mysore and Ryu, 2004; Lee et al., 2017). Previous studies have demonstrated that NLPs from various fungi, bacteria, and oomycetes act as potent activators of PTI in plants (Oome et al., 2014; Oome and Van den Ackerveken, 2014). Further, they have shown that a 24 aa peptide (nlp24) from a conserved region in the NPP1 domain is essential for triggering immune responses. The 24 aa peptide region is highly conserved and is retained in its functional form in AbrNLP1 and AbrNLP2. Recent studies in *Arabidopsis* have shown that RLP23 contributes to resistance against *B. cinerea via* recognition of BcNLP1 and BcNLP2 (Ono et al., 2020). However, they also showed that RLP23 is not essential for resistance against *Alternaria brassicicola*, since the NLP from *A. brassicicola* is not expressed at early stages of infection. Therefore, there may be other RLP proteins that may be responsible for the recognition of NLPs from the *Alternaria* species.

In conclusion, we identified two NLPs from *A. brassicae* and functionally characterized their necrosis inducing ability. AbrNLP1 and AbrNLP2 are the first NLPs from the *Alternaria* genus to be functionally characterized. We showed that AbrNLP2 could induce necrosis and PTI effectively in both host and non-host species. Our work has shown that AbrNLPs are functionally and spatially (localization) distinct and may play different but important roles during the pathogenesis of *A. brassicae*.

## Data Availability Statement

The data presented in the study are deposited in the NCBI GenBank repository, accession numbers: MZ783062 and MZ783063.

## Author Contributions

SR conceived and designed the study with inputs from TS. DD and RJ performed the cloning and transient expression experiments. SG contributed to the cloning experiments and carried out the peroxidase assays. DD performed the protein expression and purification experiments with inputs from RS. SR wrote the first draft of the manuscript. All authors contributed to the revision of the subsequent versions and approved the final manuscript.

## Conflict of Interest

The authors declare that the research was conducted in the absence of any commercial or financial relationships that could be construed as a potential conflict of interest.

## Publisher’s Note

All claims expressed in this article are solely those of the authors and do not necessarily represent those of their affiliated organizations, or those of the publisher, the editors and the reviewers. Any product that may be evaluated in this article, or claim that may be made by its manufacturer, is not guaranteed or endorsed by the publisher.
